# Band-Gap Energy and Electronic *d–d* Transitions of NiWO_4_ Studied under High-Pressure Conditions

**DOI:** 10.1021/acs.jpcc.3c03512

**Published:** 2023-07-26

**Authors:** Daniel Errandonea, Fernando Rodriguez, Rosario Vilaplana, David Vie, Siddhi Garg, Bishnupriya Nayak, Nandini Garg, Jaspreet Singh, Venkatakrishnan Kanchana, Ganapathy Vaitheeswaran

**Affiliations:** † Departamento de Física Aplicada-ICMUV, MALTA Consolider Team, Universidad de Valencia, Edificio de Investigación, Carrer del Dr. Moliner 50, Burjassot, 46100 Valencia, Spain; ‡ DCITIMAC, MALTA Consolider Team, Facultad de Ciencias, Universidad de Cantabria, 39005 Santander, Spain; § Centro de Tecnologías Físicas, Universitat Politècnica de València, 46022 Valencia, Spain; ∥ Institut de Ciència dels Materials de la Universitat de València, Apartado de Correos 2085, E-46071 València, Spain; ⊥ High-Pressure and Synchrotron Radiation Physics Division, Bhabha Atomic Research Centre, Trombay, Mumbai 400085, India; # Homi Bhabha National Institute, Anushaktinagar, Mumbai 400094, India; ∇ Department of Physics, Indian Institute of Technology Hyderabad, Kandi, 502284 Sangareddy, Telangana, India; ○ School of Physics, University of Hyderabad, Prof. C. R. Rao Road, Gachibowli, Hyderabad 500 046 Telangana, India

## Abstract

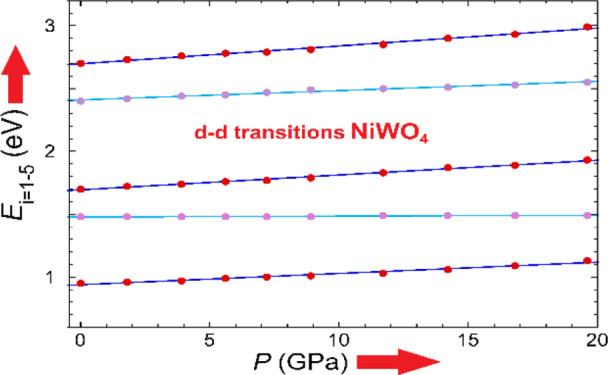

We report an extensive study of the optical and structural properties of NiWO_4_ combining experiments and density functional theory calculations. We have obtained accurate information on the pressure effect on the crystal structure determining the equation of state and compressibility tensor. We have also determined the pressure dependence of the band gap finding that it decreases under compression because of the contribution of Ni 3*d* states to the top of the valence band. We report on the sub-band-gap optical spectrum of NiWO_4_ showing that the five bands observed at 0.95, 1.48, 1.70, 2.40, and 2.70 eV correspond to crystal-field transitions within the 3*d*^8^ (*t*_2g_^6^*e*_g_^2^) configuration of Ni^2+^. Their assignment, which remained controversial until now, has been resolved mainly by their pressure shifts. In addition to the transition energies, their pressure derivatives are different in each band, allowing a clear band assignment. To conclude, we report resistivity and Hall-effect measurements showing that NiWO_4_ is a *p*-type semiconductor with a resistivity that decreases as pressure increases.

## 1 Introduction

Because of global warming the development of renewable energy sources and environmentally friendly energy storage is a hot topic. Photocatalytic water splitting and supercapacitors are two of the most promising technologies. Nickel tungstate (NiWO_4_) has been studied as an efficient material for both technologies.^1,2^ This and other tungstates are also extraordinary scintillator materials for high-energy physics.^3^ The accurate understanding of the properties of NiWO_4_ is fundamental for the above-mentioned technological applications. NiWO_4_ crystallizes in a monoclinic structure described by space group *P*2/*c*.^4^ It is isomorphic to the mineral wolframite - (Fe,Mn)WO_4_ –^5^ sharing the structure with several tungstates, including CoWO_4_, CdWO_4_, MgWO_4_, MnWO_4_, and ZnWO_4_.^6^ High-pressure studies have contributed to the understanding of the structural and electronic properties of wolframites.^7−10^ High pressure (HP) can substantially modify the structural and electronic properties of materials favoring a deep understanding of them.^11^ NiWO_4_ is one of the wolframites that has been less studied under compression, there being only one article recently published.^10^ In this work, a phase transition was reported around 20 GPa, which is in consistent with results previously reported for other wolframites.^6^ In addition, the pressure dependence of the band-gap energy (*E*_gap_) and two Ni^2+^*d–d* intra-band transitions were reported. However, there are several issues regarding the electronic properties of NiWO_4_, and their HP behavior that still needs to be clarified. For instance, the assignment of Ni^2+^*d–d* transitions made by Ye et al.^10^ from measurements carried out in the 1.45–3.5 eV region is at odds with the assignment made by Ejima et al.^12^ and by de Oliveira et al.^13^ from experiments performed in the 0.82–6.0 eV region and in the 1.3–6.5 eV region, respectively. On the other hand, for the band-gap energy, a value of 2.87 eV has been reported in the most recent work.^10^ However, a large dispersion of band-gap energies can be found in the literature for NiWO_4_. For instance, *E*_gap_ = 3.4 eV was reported in ref (12), *E*_gap_ = 3.7 eV in ref (13), *E*_gap_ = 2.73–2.93 in ref (14), *E*_gap_ = 2.1 eV in ref (15), *E*_gap_ = 2.2–2.6 eV in ref (16), *E*_gap_ = 2.77 eV in ref (17), and *E*_gap_ = 2.28 eV in ref (18). These results clearly show that it is timely to perform additional studies on NiWO_4_. Photocatalytic water splitting and photocatalytic wastewater treatment require semiconductors with the band-gap energy in the visible part of the solar spectrum, in particular with values from 2.0 to 2.6 eV.^19^ The accurate knowledge of the band-gap energy is therefore crucial for the use of NiWO_4_ for these applications.

In this work, we report a HP study of NiWO_4_ up to 20 GPa. Our study includes HP synchrotron X-ray diffraction (XRD) experiments and optical-absorption experiments in the 0.82–3.6 eV region up to 20 GPa, resistivity and Hall-effect measurements up to 10 GPa, and density-functional theory (DFT) calculations up to 20 GPa. The combination of all the methods allows us to accurately determine the electronic properties of NiWO_4_ and the effect of pressure on them up to 20 GPa. We show that changes in the fundamental band gap and Ni^2+^ internal *d–d* absorption bands are intimately related to structural changes. The assignment of Ni^2+^*d–d* transitions is discussed, clarifying previous discrepancies in the literature.

## 2 Methods

Polycrystalline NiWO_4_ has been synthesized from a stoichiometric mixture of nickel(II) acetate hexahydrate Ni(CH_3_CO_2_)_2_· 6H_2_O (Fluka, 98%) and ammonium metatungstate (NH_4_)W_12_O_39_ (Aldrich, 99.0%). The starting Ni- or W-containing solutions were prepared by dissolving their respective salts in distilled water. Then they were combined to obtain Ni-W source solutions having a total cationic concentration of 0.3 M and a total volume of 21.7 mL. The masses of the different reagents were adjusted to obtain 1 g of the final product. Droplets of the solution were flash-frozen by projection onto liquid nitrogen and then freeze-dried at a pressure of 1–10 Pa in a Telstar Cryodos freeze-dryer. In this way, a dried solid precursor was obtained as amorphous loose powder. Thermal evolution of the precursor was monitored by means of thermogravimetric experiments under an oxygen atmosphere (heating rate 10 K min^–1^, flow rate 60 cm^3^ min^–1^), carried out using a TA Instruments TG550 thermogravimetric analyzer. The final NiWO_4_ polycrystalline product was obtained by decomposing the precursor in an oven at 800 °C for 1 h. The purity and crystal structure of the synthesized NiWO_4_ was confirmed by powder XRD using a Bruker D8 Advance A25 system and Cu K_α_ radiation. We have not detected any impurity in the XRD experiments. The unit-cell parameters at ambient conditions are *a* = 4.599(3) Å, *b* = 5.664(5) Å, *c* = 4.910(5) Å, β = 90.06(7)°. These values agree with those determined from single-crystal neutron diffraction experiments.^20^

HP synchrotron powder XRD measurements were carried out at the Extreme Conditions XRD beamline (BL-11)^21^ in the Indus-2 synchrotron using monochromatic X-rays (λ = 0.731 Å) and a diamond-anvil cell (DAC). We used diamond anvils with a culet size of 400 μm and a tungsten gasket with a thickness of 50 μm with a centered hole of 200 μm in diameter. The pressure medium was a 16:3:1 methanol–ethanol–water mixture (MEW)^22^ and copper was used as a pressure marker.^23^ Diffraction images were collected in a MAR345 imaging-plate detector. Their integration into intensity vs 2θ patterns was made using Dioptas.^24^ The XRD results patterns were analyzed using the graphical interface of GSAS.^25^

Optical-absorption measurements were carried out in a DAC using a 3-μm-thin platelet of NiWO_4_ obtained by compacting NiWO_4_ powder between diamond anvils. The characteristics of the DAC were the same as in XRD measurements. The pressure medium was also the same, and the gasket had similar dimensions but was made of Inconel. Pressure was determined using the ruby fluorescence method.^26^ Measurements in the 0.82–3.6 eV range were performed in an optical setup, which consisted of a tungsten lamp, fluorite lenses, reflecting optics objectives, and several Ocean Optics spectrometers.^27^

Hall-effect and resistivity measurements were performed with a hydraulic press and steel-belted Bridgman tungsten-carbide anvils^28^ using annealed pyrophyllite gaskets and boron nitride as pressure medium and to electrically isolate the sample from the anvils. Samples were compact pellets made from the NiWO_4_ powder. Electrical contacts were made with silver wires and soldered with indium to the sample in a Van der Pauw configuration. The pressure was calibrated against the transition pressures of Bi, Yb, CdTe, and InSe.^29^

The *Vienna Ab-initio Simulation Package* (VASP) code,^30,31^ which is based on plane waves, was used to study the electronic and structural characteristics of NiWO_4_ at HP. The exchange correlation functionals were computed using the Perdew–Burke–Ernzerhof (PBE) potentials within the Generalized-Gradient Approximation (GGA),^32^ and Projector augmented wave (PAW)^33^ potentials were used to approximate the ion-electron interactions. In calculations we treated Ni 3*d*^8^ 4*s*^2^, W 6*s*^2^ 5*d*^4^, and O 2*s*^2^ 2*p*^4^ explicitly as valence electrons and the rest of electrons as core electrons. To ensure accuracy and high precision in the calculations, we used a plane-wave energy cutoff of 600 eV. The energy convergence criteria have been chosen to be 10^–6^ eV. The geometry optimization calculations have been performed at 0 K by doubling the unit-cell along the *a*-axis to consider magnetic configurations and using a 4 × 6 × 8 k-mesh in accordance with the Monkhorst–Pack method.^34^ According to the various investigations of transition metal compounds, the GGA frequently produces the wrong findings in case of highly correlated systems. In this case, the inclusion of Hubbard parameter (*U*) has been found to have some impact on transition metal compounds.^35^ The significant correlation effects of nickel *d* states were treated by using a Hubbard (*U*) parameter (GGA + *U*) of 6.5 eV.^36^ Because NiWO_4_ presents a magnetic moment, we considered non-magnetic, ferromagnetic, and different antiferromagnetic configurations.^37^ We found that the configuration with the lowest energy is an antiferromagnetic order with a magnetic unit cell (2*a*, *b*, *c*) which doubles the crystallographic one along the *a*-axis. The spins at nickel atoms are arranged collinearly making a linear chain running nearly along the crystallographic [0,1̅,1] direction, but are antiparallel to the spins in adjacent chains. This configuration agrees with the magnetic configuration determined below 67 K from neutron diffraction experiments.^38^ After optimizing the crystal structure at different pressure, band structures, and electronic densities of states have been calculated. We have also determined the complex dielectric function which has been used to calculate the absorption coefficient by means of the Kramers–Kronig relationship.^39^

## 3 Results and Discussions

### 3.1 XRD Experiments

**Figure 1:**
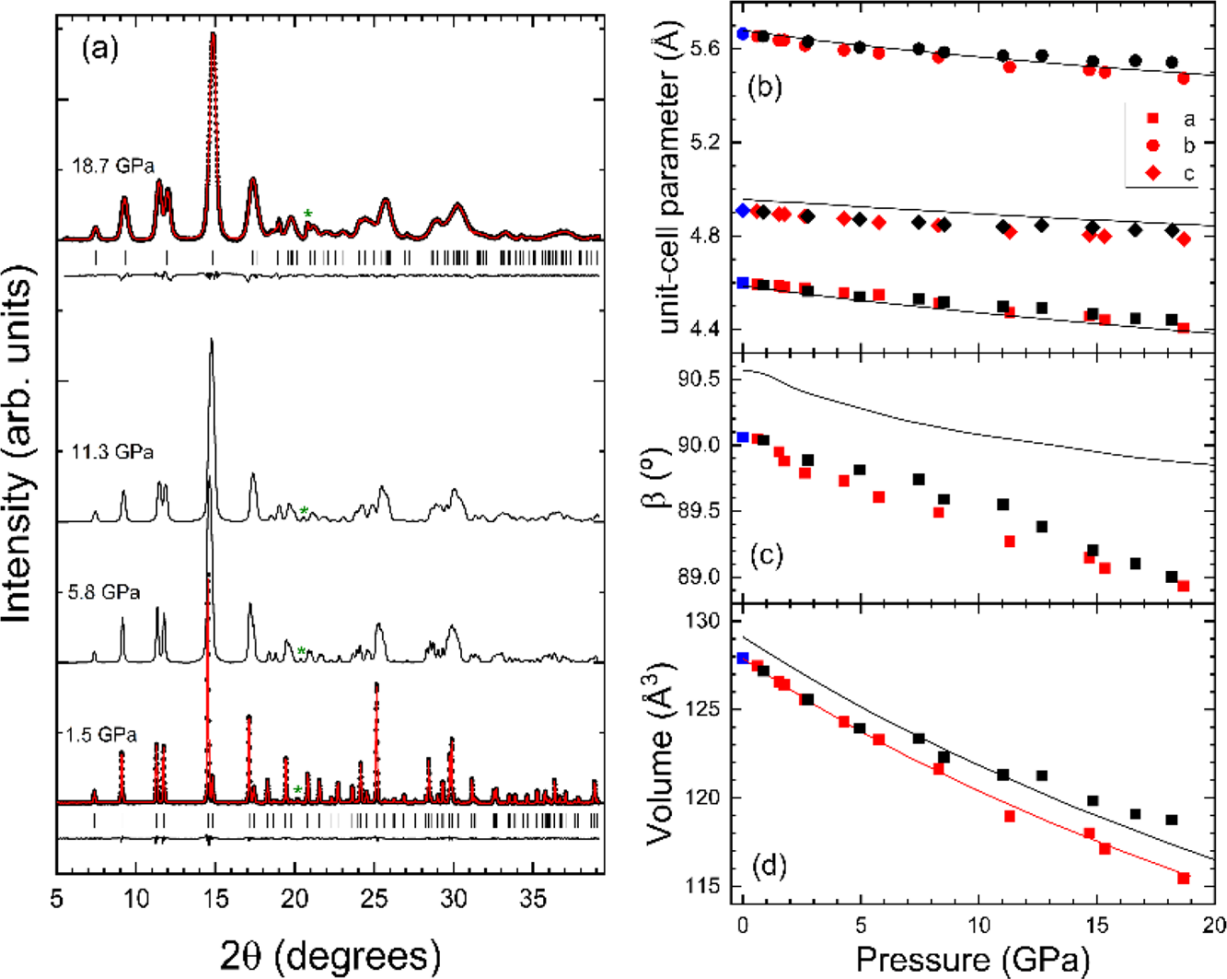
(a) XRD patterns measured at different pressures which are indicted in the figure. In the top and bottom traces, we show experiments (black dots), refinements (red lines), residuals (black lines), and positions of reflections (vertical ticks). The green asterisks identify the peak of Cu used to determine pressure. (b) Pressure dependence of the lattice parameters *a*, *b*, and *c*. (c) Pressure dependence of the β angle. (d) Pressure dependence of the unit-cell volume. Blue symbols are from our experiment performed outside the DAC. Red symbols are from our HP experiments, and black symbols are from ref (10). The black lines are from our DFT calculations, and the red line represents the 3rd order Birch–Murnaghan EOS determined from our experiments.

In Figure 1a, we present a selection of XRD patterns measured at different pressures. In agreement with ref (10), we have found that NiWO_4_ does not undergo any phase transition in the pressure range covered by our studies, i.e., the crystal structure can be described as isomorphic to wolframite up to 20 GPa. This is supported by the results of Rietveld refinements shown in Figure 1a at 1.5 and 18.7 GPa. The goodness-of-fit parameters are *R*_p_ = 3.46% and *R*_wp_ = 5.38% at 1.5 GPa (*R*_p_ = 7.42% and *R*_wp_ = 10.53% at 18.7 GPa). The broadening of peaks observed at 18.7 GPa is a consequence of the solidification of the pressure medium at 10 GPa and the consequent increase of pressure gradients across the pressure chamber.^22^ The observed peak broadening is typical in experiments performed using 16:3:1 MEW as pressure-medium and has also been observed in ref (10).

From the structural refinements, we have obtained the pressure dependence of unit-cell parameters. The results are shown in Figure 1b–d. In the figures, we compare the results of our experiments with the previous experiments^10^ and our DFT calculations. All results show a similar pressure dependence with the calculations slightly overestimating the value of the β angle. Interestingly the previous experiments^10^ show an abrupt decrease of the volume compressibility around 7 GPa. This phenomenon is not observed in our experiments and calculations. The change of compressibility could not be related to non-hydrostatic effects because the present and the previous experiment were performed using the same pressure medium, which is quasi-hydrostatic up to 10 GPa. We think the change of compressibility might be related to sample bridging between diamonds, which could strongly affect the compressibility of the sample.^40^

We have used a third-order Birch–Murnaghan equation of state^41^ to fit the pressure dependence of the volume obtained from our experiments. The zero-pressure volume (*V*_0_), bulk modulus (*K*_0_), and its pressure derivative (*K*_0_′) we have determined are *V*_0_ = 128.0(3) Å^3^, *K*_0_ = 137(17) GPa, and *K*_0_′ = 5.5(2.3). From our calculations we have obtained *V*_0_ = 129.12(1) Å^3^, *K*_0_ = 147.4(6) GPa, and *K*_0_′ = 5.38(8). The values of *K*_0_ and *K*_0_′ obtained from our experiments and calculations agree within uncertainties, which shows that calculations accurately describe the changes induced by pressure in the crystal structure. In the previous study,^10^ it was reported that according to experiments, *V*_0_ = 129.12(1) Å^3^, *K*_0_ = 146.05(2) GPa, and *K*_0_′ = 14.40(1). We consider that the overestimation of the pressure derivative of the bulk modulus is an artifact caused by the abrupt change of compressibility that occurs in previous experiments at 7 GPa.^10^ From previous calculations, it was reported that *V*_0_ = 133.16(8) Å^3^, *K*_0_ = 162.91(2) GPa, and *K*_0_′ = 3.64(2). The bulk modulus is larger than in our present results, but the pressure derivative is smaller. Thus, since both parameters are correlated,^42^ it is not obvious to compare results from previous calculations and the previous study. To do it, we have refitted the results of previous calculations, but fixing *K*_0_′ = 5.38, the values obtained from our calculations. This way we have obtained *V*_0_ = 133.39(8) Å^3^ and *K*_0_ = 147.3(1.5) GPa in excellent agreement with our results. Therefore, we are confident that the bulk modulus of NiWO_4_ is 137–147 GPa, and its pressure derivative is 5.4–5.5.

From Figure 1b, it can be seen that the compressibility of NiWO_4_ is slightly anisotropic. Since the crystal structure is monoclinic, the analysis of compressibility is not straightforward and requires the use of the compressibility tensor, which has four elements different than zero,^43^ and the determination of its eigenvalues and eigenvectors. We have obtained them using PASCal.^44^ We have found that the main axes of compressibility are (010), (8̅09), and (806). The corresponding linear compressibilities are 2.6(1) × 10^–3^ GPa, 2.0(2) × 10^–3^ GPa, and 1.3(1) × 10^–3^ GPa, respectively. As in other wolframites, the most compressible axis is the *b*-axis. The other two axes have similar compressibilities. They are in the plane perpendicular to the *b*-axis making an angle of 86° between them.

### 3.2 Optical-Absorption Experiments

**Figure 2:**
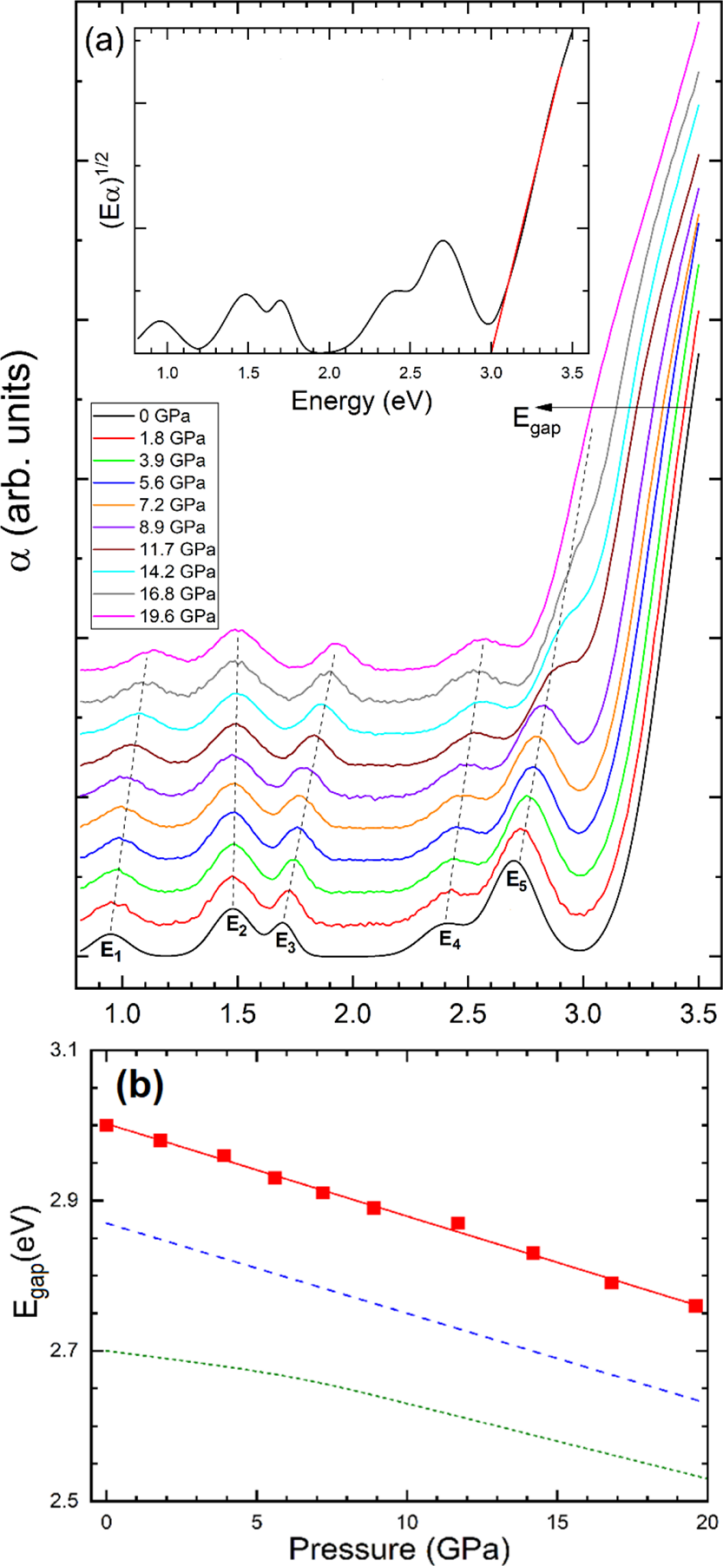
(a) Absorbance (α) of NiWO_4_ at different pressures. The spectra have been offset vertically to make their identification easier. The assignment for the sub-gap bands is provided at 0 GPa. The dashed lines follow the sub-gap bands with pressure increase. The inset shows the Tauc plot at 0 GPa with the solid line used to determine the band-gap energy, *E*_gap_ = 3.0 eV. (b) *E*_gap_ determined from experiments (symbols), present calculations (dotted line), and from ref (10) (dashed line). The red solid line is a linear fit to experiments.

In Figure 2a, we show the results of our optical-absorption measurements at different pressures. At 0 GPa, there is a sharp absorption at high-energy that corresponds to the fundamental band gap and five absorption bands that correspond to sub-gap Ni^2+^*d–d* internal transitions which are identified as *E_i_* (*i* = 1–5). As pressure increases, the band gap red-shifts (see Figure 2a) and four of the Ni^2+^*d–d* bands blue-shift, while the fifth one does not move with pressure. Consequently, the Ni^2+^*d–d* band which is at 2.7 eV at 0 GPa moves into the fundamental band gap. To determine the band-gap energy, we have used a Tauc plot^45^ assuming an indirect band gap (as supported by our DFT calculations which are reported at the end of this subsection). In this method, the band-gap energy is determined from the extrapolation to the abscissa of the linear region of (*E*α)^1/2^ versus energy (see Figure 2a). Readers should be aware that this energy should be considered as a lower bound for the band-gap energy.^46^ As it can be seen in the inset of Figure 2a, the band-gap energy at 0 GPa is estimated to be 3.00(5) eV. Comparing with previous experiments,^10,12−18^ our band-gap energy best agrees with the result reported by Ye et al.^9^ From our experiments, we have determined the pressure dependence of the band-gap energy (*E*_gap_) which is shown in Figure 2b. The pressure dependence of *E*_gap_ can be well described by a linear function as shown with a red solid line in Figure 2b. The pressure coefficient of this function, d*E*_gap_/d*P*, is −13(1) meV/GPa, which is in excellent agreement with previous results.^10^ The pressure dependence obtained from experiments also agrees with that obtained from our calculations (see Figure 2b).

**Figure 3:**
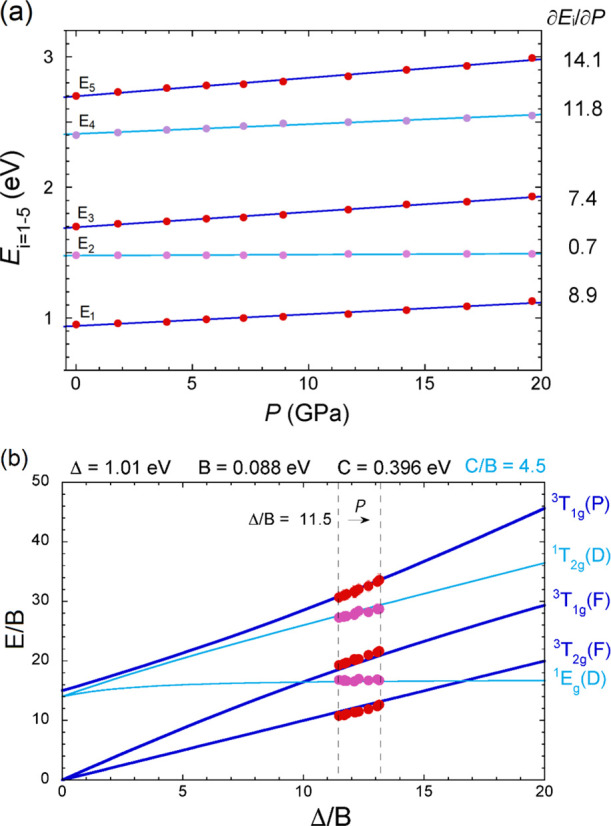
(a) Pressure dependence of the energy of Ni^2+^*d–d* transitions. Symbols are from the present experiment. The solid lines are from present pressure fits collected in Table 1. The pressure shift of each band is given right side in meV/GPa units. (b) Tanabe–Sugano (TS) diagram used for the interpretation of experiments showing the variation of the crystal-field transition energies, *E*/*B*, as a function of Δ/*B* within the *d*^8^ configuration of Ni^2+^. Circles are experimental data obtained at different pressures. In both figures, dark blue and cyan curves represent results for the spin-allowed transitions and for transition with spin change, respectively.

From the experiments, we could also follow the pressure dependence of Ni^2+^*d–d* transitions (see dashed lines in Figure 2a). The results are shown in Figure 3a. We have determined the pressure dependence for the five Ni^2+^*d–d* bands. In ref (10), only the pressure dependence of bands here labeled as *E*_3_ and *E*_5_ was reported. For these two bands, we observed the same linear increase of the energy with pressure. In our case, we obtained d*E*_5_/d*P* = 14.8 meV/GPa and d*E*_3_/d*P* = 11.8 meV/GPa, while the values reported for the pressure coefficients in ref (10) are 14.8 and 7.4 meV/GPa, respectively. In Figure 3a, it can be also seen than *E*_1_ and *E*_4_ increase under compression, whereas *E*_2_ is not affected by pressure. A detailed behavior of the pressure dependence of Ni^2+^*d–d* transitions will be discussed in the next section.

Interestingly, in NiWO_4_, the band gap closes under compression as observed in isomorphic MnWO_4_,^6^ CoWO_4_,^7^ and CuWO_4_.^8^ On the other hand, this behavior is the opposite to what happens in wolframite-type CdWO_4_, ZnWO_4_, and MgWO_4_^6^ in which the band gap opens under compression. This latter behavior is a result of the increase of the crystal field (CF) which enhances the splitting between the top of the valence band and the bottom of the conduction band which are dominated by O *2p* states and W *5d* states. As in MnWO_4_, CoWO_4_, and CuWO_4_, it is possible that the closing under pressure of the band gap in NiWO_4_ could be related to the contribution of Ni *3d* states to the top of the valence band or the bottom of the conduction band. This hypothesis is supported by our DFT calculations as we will show next.

**Figure 4:**
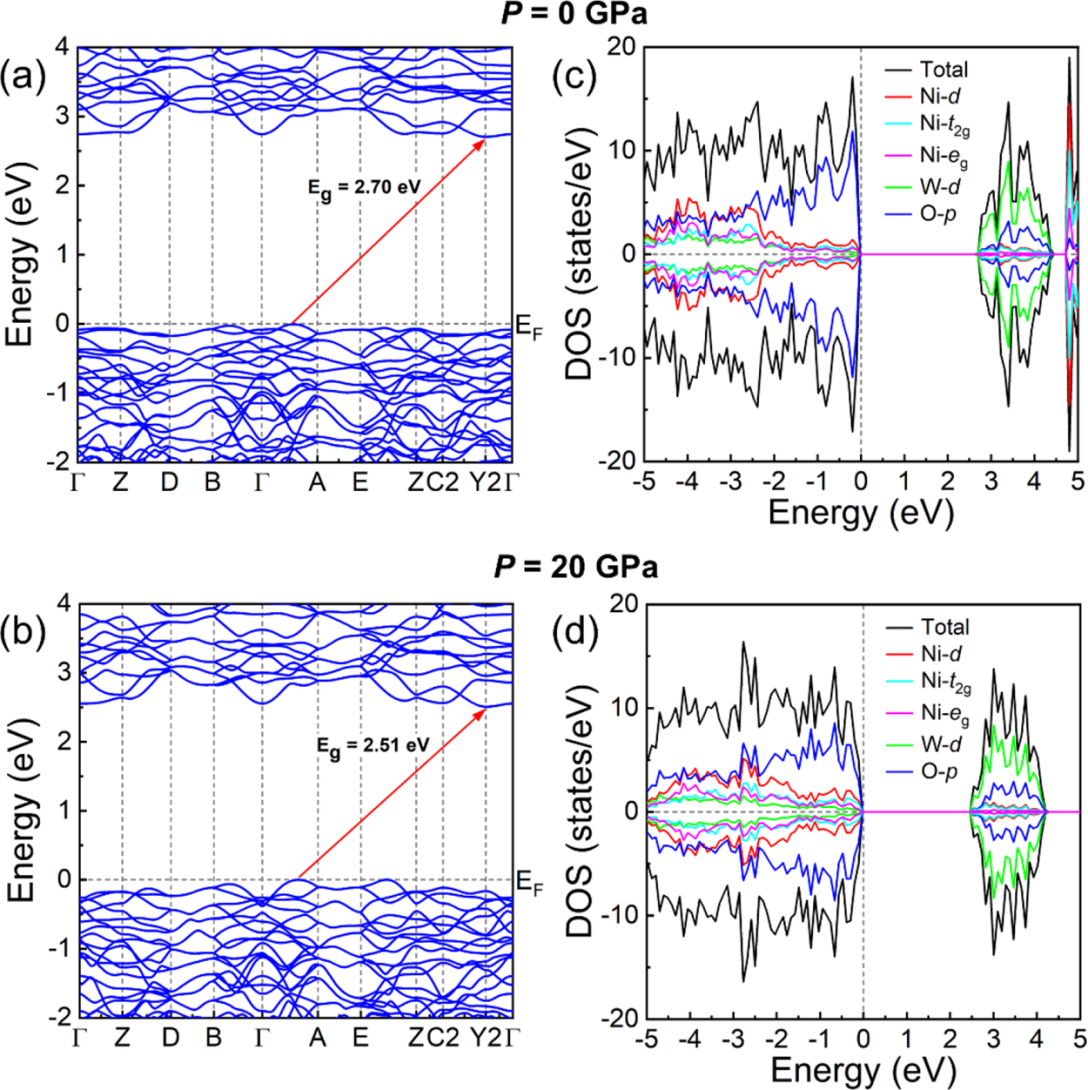
(a) Band structure at 0 GPa. (b) Band structure at 20 GPa. (c) Electron density of states at 0 GPa. (d) Electron density of states at 20 GPa.

In Figure 4, we show the calculated band structure (4a and 4b) and electron density of states (4c and 4d) at 0 and 20 GPa. Previous calculations gave a band-gap energy of 2.1 eV^10^ and 3.91 eV^47^ at 0 GPa. Our calculations give a band-gap energy of 2.7 eV, which is much closer to the experimental value. Calculations by Ye et al.^10^ might have underestimated the band gap because they consider a non-magnetic configuration and/or used a Hubbard parameter *U* = 3.2 eV for Ni. We carried out simulations for the magnetic stable configuration using the same Hubbard parameter and obtained a band-gap energy of 2.07 eV showing that the problem of previous calculations was mainly the choice of the Hubbard parameter. On the other hand, Rosal et al.^47^ performed calculations using the Becke 3-paramters Lee–Yang–Parr (B3LYP) hybrid functional^48^ assuming a non-magnetic configuration and ignoring the Hubbard term. In the Supplementary Material of their work, it can be seen that this approach does not describe as accurately as our calculations the crystal structure of NiWO_4_ at 0 GPa. Thus, it is not surprising that their DFT approach leads to an overestimated band-gap energy.

**Figure 5:**
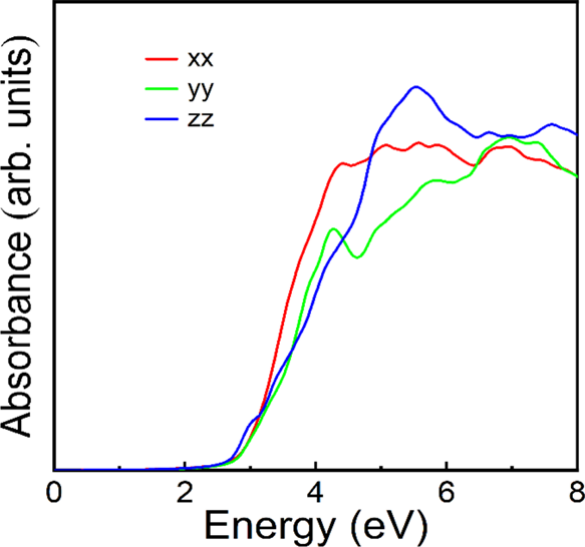
Calculated absorbance spectrum at 0 GPa for different polarizations.

**Figure 6:**
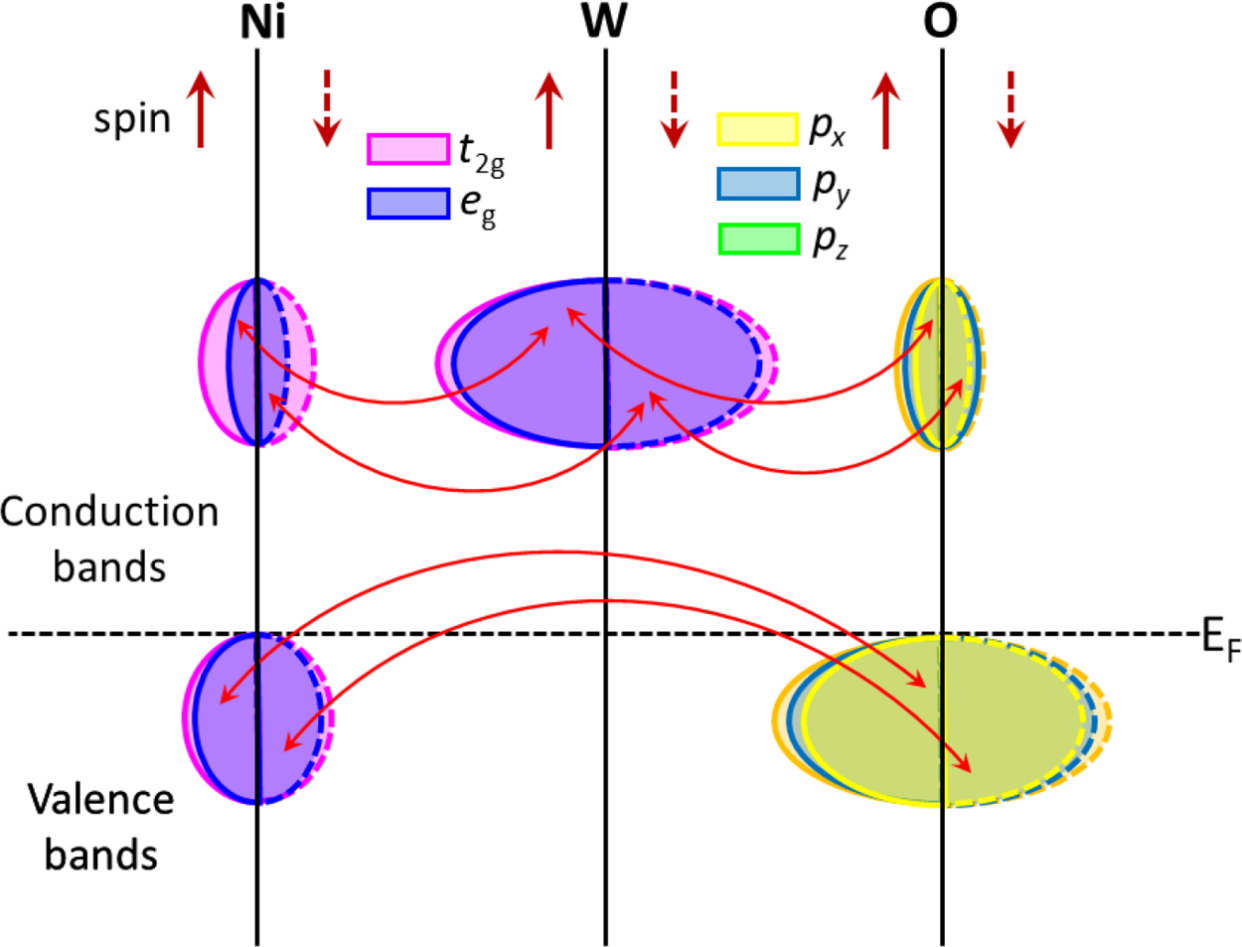
Schematic representation of the electronic density of states of NiWO_4_ around the Fermi level.

The band structure at 0 and 20 GPa is represented in Figure 4a,b. The electron density of states at 0 and 20 GPa is represented in Figure 4c,d. We have also calculated the absorption coefficient which is shown in Figure 5. Figure 4 shows that NiWO_4_ is an indirect band gap semiconductor and that the top of the valence is dominated by O *2p* states with a minor contribution from Ni *3d* states. Contrastingly, the bottom of the conduction band is dominated by W *5d* states. A schematic representation of the electronic density of states around the Fermi energy is shown in Figure 6. As expected for a compound with a conduction band with *d* character, the conduction is not very dispersive, and its energy minimum is not in the zone center being at the Y2 point of the Brillouin zone (see Figure 4a). On the other hand, the top of the valence band is at a point near the A point in the Brillouin zone. Regarding the absorption-coefficient, calculations show that NiWO_4_ has an abrupt absorption starting at 2.7 eV (see Figure 5), which agrees with the sharp absorption edge found in the experiments performed by Ejima et al. at 3.0 eV. Indeed, the shape of measured and calculated absorption is qualitatively similar. However, calculations do not predict the sub-bandgap Ni^2+^*d–d* transitions because they are not accounted by the electric dipole approximation. In order to accurately calculate *d*–*d* transitions occurring below the band gap, it may be necessary to employ alternative methodologies that go beyond the electric dipole approximation. For example, methods such as time-dependent DFT or more advanced approaches like many-body perturbation theory (MBPT) can be utilized. These methods are capable of considering higher-order effects and providing a more precise description of the *d–d* transitions occurring below the bandgap.^49,50^ Such calculations are beyond the scope of the present work.

Regarding pressure effects on the band structure, Figure 4b shows that at 20 GPa, the topology of the band structure has not changed significantly from 0 GPa (Figure 4a). However, though the bottom of the conduction band goes down with pressure, the top of the valence band remains at the same energy due to the increase of the contribution of Ni *3d* states to it. This is translated into the band-gap energy decrease we have observed in experiments. This behavior in NiWO_4_ is qualitatively like those previously reported MnWO_4_^6^ and CoWO_4_^7^ as the contribution to band-gap energy in these compounds is also due to the contribution of *3d* states of Mn and Co near the Fermi level.

### 3.3 Ni^2+^*d–d* Transitions

The origin of the sub-band-gap absorption features in NiWO_4_ has been controversial and still deserves clarification.^10,12,13,49^ So far, all papers dealing with the optical properties of NiWO_4_ have reported different origins or misinterpretations of the sub-band-gap bands as Ni^2+^ CF transitions, defect related absorption, or excitonic components of the gap. Lima et al.^51^ as well as Ye et al.^10^ assigned bands at 1.68 and 2.73 eV to spin forbidden CF transitions from ^3^*A*_2g_ to ^1^*E*_g_ and ^1^*T*_2g_, respectively. In contrast, Ejima et al.,^12^ who reported energies similar to ours, assigned these bands to spin-allowed CF transitions with ^3^*T*_1_ symmetry after comparison with the assignment given for [Ni(H_2_O)_6_]^2+^ and NiO.^52^ Ye et al.^10^ attributed the band at 2.74 eV to an exciton associated with the band gap and the band at 1.48 eV, which does not move with pressure, to “the presence of Frenkel defects with the dislocation of Ni^2+^ from the octahedron to the tetrahedron site.” On the other hand, de Oliveira et al.^13^ assigned a shoulder at ∼2.7 eV as probably due to the presence of a midgap defect state. In all cases, the pressure experiments associated with the sub-band-gap spectra lack absorption bands, or they are poorly resolved by reflectometry measurements on NiWO_4_ powders, making a proper band assignment difficult. In this context, HP measurements are important because the associated pressure shifts provide critical information for a proper band assignment. In this work, we have used a NiWO_4_ sample sintered at HP using a DAC. This method provides a transparent parallelepipedal sample to perform suitable optical-absorption measurements at ambient conditions and as a function of pressure, which was crucial to achieve this goal.

The room-temperature optical absorption sub-gap spectrum of NiWO_4_ shows five absorption bands, named *E_i_*, at 0.95, 1.48, 1.70, 2.40, and 2.70 eV from *i* = 1 to 5, respectively. These five bands are also observed as main absorption features in the CF spectra of Ni^2+^ in oxides, fluorides, and chlorides,^52−57^ as well as in NiWO_4._^12^ The proposed band assignment to electronic CF transitions within the *3d^8^* configuration of Ni^2+^ is given in Figure 3b. Peak labels have been assigned according to the sixfold O_h_ symmetry of the NiO_6_ local environment of Ni^2+^. Although the actual local symmetry around Ni^2+^ is slightly distorted with respect to O_h_, the CF transition energies as well as their pressure dependence are well described on the basis of the TS diagram for *d^8^*(58,59) (See Figure 3b). The energy of the five measured bands and their different pressure shifts were crucial for a correct band assignment. The Supplemental Material collects the measured transition energies at each pressure and the corresponding calculated energies as a function of the Racah parameters *B* and *C* and the CF energy Δ (=10*Dq*) of the 3*d*-orbitals split into *e*_g_ + *t*_2g_. The assignment and CF parameters are similar to those originally given elsewhere^53−57^ (See Figure S1). Therefore, the sub-gap bands correspond to intra-configurational CF transitions within the 3*d*^8^ configuration from the ^3^*A*_2g_(F) [*t*_2g_^6^*e*_g_^2^] ground state to the excited states ^3^*T*_2g_(F), ^3^*T*_1g,a_(F), ^1^*E*_g_(D), ^1^*T*_2g_(D), and ^3^*T*_1g,a_(P), in order of increasing energy, in the low CF limit (Δ/*B* < 10). However, the ^3^*T*_1g,a_(F) state energy exceeds the ^1^*E*_g_(D) energy for (Δ/*B* > 10). Their transition energy as a function of *B* and *C* and Δ (see Figure 3b) is given by^58,59^
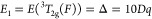

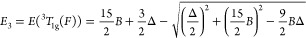

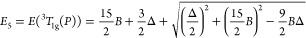
1for the spin-allowed transitions, and by
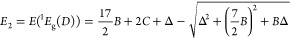

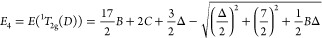
2for transitions with spin change.

In particular, the energy of the ^3^*A*_2g_(*F*) → ^1^*E*_g_(*D*), *E*_2_, is practically independent of Δ for Δ/*B* > 5. The TS for Ni^2+^ as a plot of *E*/*B* vs Δ/*B* can be obtained directly from eqs 1 and 2 for a given *C*/*B* ratio. The energy of the ^3^*A*_2g_(*F*) → ^1^*E*_g_(*D*) transition, which corresponds to a spin-flip transition within the *t*_2g_^6^*e*_g_^2^ configuration, runs parallel to the Δ/*B* abscissa axis in the TS diagram for about Δ/*B* > 5 (Figure 3b). The parameters *B* and Δ at ambient pressure were obtained by least-squares fitting of the experimental energies *E*_1_, *E*_3_, and *E*_5_, whose energies depend on *B* and Δ but not on *C*, to eq 1. On the other hand, the spin-flip transition energies *E*_2_ and *E*_4_, which both depend on *B*, *C*, and Δ, were used to determine the parameter *C* from eq 2. The TS diagram of Figure 3b shows the experimental CF transition energies of the NiWO_4_ absorption spectrum and the calculated ones using *B* = 0.088 eV, Δ = 1.01 eV; *C*/*B* = 4.5 (Δ/*B* = 11.5). Comparisons between measured and calculated energies at ambient pressure and as a function of pressure are collected in Table S1 at SI. It should be noted that the values obtained for *B*, *C*, and Δ at ambient pressure are somewhat unusual compared to other oxides and fluorides.^53−57^ For example, the spectrum of NiWO_4_ looks similar to the Ni^2+^ in MgO (bunsenite) or NiO (periclase) where energies of the spin-allowed transitions appear at 1.07 eV (^3^*T*_2g_(*F*)), 1.67 eV (^3^*T*_1g_(*F*)), and 3.05 eV (^3^*T*_1g_(*P*)); and 1.09, 1.75 and 2.99 eV, respectively;^53,54^ or 0.91, 1.55, and 2.95 eV in KNiF_3._^55^ It must be noted that the separation between the experimental ^4^*T*_1g_ energies, *E* = *E*(^3^*T*_1g_(*P*))- *E*(^3^*T*_1g_(*F*)) = *E*_5_ – *E*_3_, which has values of 1.38 eV for MgO, 1.24 eV for NiO, and 1.40 eV for KNiF_3_, is only of 1.0 eV for NiWO_4_ having a similar Δ value. This relatively smaller energy difference between *E*_5_ and *E*_3_ reflects the lower value of the measured CF splitting Δ = 0.95 eV as compared to that obtained by fitting Δ = 1.01 eV (about 6%). The opposite trend is observed for *E*_3_ (see Table S1 in Supplementary Material).

An examination of the optical absorption of NiWO_4_ at ambient pressure suggests that the second band *E*_2_ could initially be assigned to ^3^*T*_1g_(*F*) and the third band *E*_3_ to ^1^*E*_g_(*D*). In fact, such an assignment is in better agreement with the transition energies of other Ni^2+^ compounds with CF splitting lower than 1.0 eV (*E*_1_ < 1.0 eV) such as KNiF_3_^54^ than with Ni^2+^ oxides with CF values of about 1.2 eV (*E*_1_ > 1.0 eV) obtained in NiO and other oxides.^53−55^ In addition, the assignment of *E*_3_ to ^1^*E*_g_(*D*) would be supported by the fact that its intensity is lower than *E*_2_ as it would be expected for a spin-flip transition such as ^1^*E*_g_(*D*) as opposed to a spin-allowed transition such as ^3^*T*_1g_(*F*). The assignment given elsewhere^10,51^ is consistent with this interpretation. However, the fact that we are close to the crossover of the ^3^*T*_1g_(*F*)-^1^*E*_g_(*D*) states around Δ/*B* = 10 makes the band assignment more complicated. Under such circumstances, the Fano resonance induced by the spin-orbit coupling interaction at the crossover point can equal their respective absorption coefficient, as occurs at the ^4^*T*_2g_ – ^2^*E*_g_ crossover point in Cr^3+^.^60^ Furthermore, the electric-dipole exchange-induced mechanism, which is very active in exchange-coupled Ni^2+^ pairs or concentrated materials,^56,57^ can modify the absorption intensities of spin-flip transitions.

Pressure measurements definitely clarify the band assignment based on the pressure shifts. Besides the transition energies *E_i_*, the pressure derivatives  are very different for *E*_2_ and *E*_3_ as it is shown in the TS diagram of Figure 3b. Furthermore, we can directly compare the measured pressure shifts  and the calculated CF-derivatives of *E_i_*, , as derived from eqs 1 and 2. These two derivatives are related by the pressure derivative of the CF, , which for Ni^2+^ coincides with the pressure derivative of the first band:  = .

Therefore,  and  are related by the expression:  =  = . So that, the experimental variation  can be obtained from the pressure shifts through the expression: .

The variations *E_i_*(*P*) are shown in Figure 3a together with their respective pressure derivative . Table 1 compares the experimental pressure derivatives  with the calculated ones  from eqs 1 and 2 for Δ/*B* = 11.5. It turns out that, as in most oxides, the energy of the CF-independent spin-flip transition ^1^*E*_g_(*D*) corresponds to *E*_2_, while ^3^*T*_1g_(*F*) to *E*_3_. The assignment of *E*_1_, *E*_4_, and *E*_5_ to ^3^*T*_2g_(*F*), ^1^*T*_2g_(*D*) and ^3^*T*_1g_(*P*), respectively, is consistent with both their energy and CF-derivative, thus clarifying the origin of the present sub-gap bands in NiWO_4_. Figure 3b shows the TS diagram and the measured energies with respect to the *B* parameter. The agreement between the measured and calculated energies with Δ/*B* is fully consistent with the proposed band assignment.

**Table 1:** Transition Energies and Corresponding Pressure Shifts of the Five Sub-Gap Bands *E_i_* (*i* = 1–5) of NiWO_4_ at Ambient Pressure^a^

transition energy	*E*_1_	*E*_2_	*E*_3_	*E*_4_	*E*_5_
band assignment ^3^*A*_2g_(*F*)→	^3^*T*_2g_(*F*)	^1^*E*_g_(*D*)	^3^*T*_1g_(*F*)	^1^*T*_2g_(*D*)	^3^*T*_1g_(*P*)
experimental *E_i_* (*P* = 0; eV)	0.95	1.48	1.70	2.40	2.70
experimental (meV GPa^–1^)	8.9	0.7	11.8	7.4	14.1
experimental	1.0	0.08	1.33	0.83	1.62
calculated from TS	1.0	0.04	1.40	1.07	1.60

a The proposed band assignment to crystal-field transitions within the 3*d*^8^ configuration of Ni^2+^ is included. The comparison between experimental and calculated crystal-field derivatives of the transition energies are shown in last two rows. The crystal-field derivatives were calculated from eqs 1 and 2 for Δ/*B* = 11.5.

**Figure 7:**
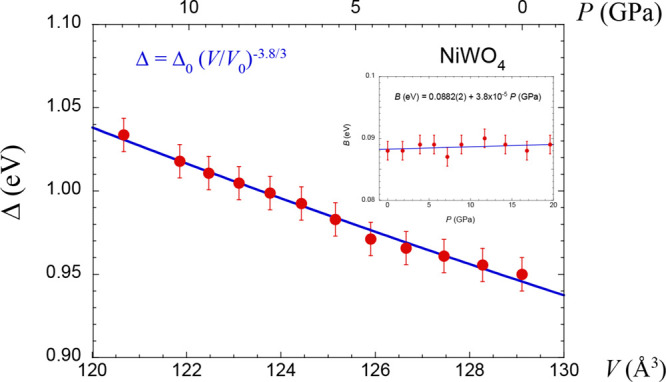
Variation of the CF splitting Δ(*P*) with the crystal volume *V* using the *V*(*P*) data of Figure 1b. The Δ(*V*) data have been fitted to the equation with fit parameters of *n* = 3.8(4), Δ_0_= 0.946(2) eV, and *V*_0_= 129.1(2) Å^3^. The inset is the variation *B*(*P*) in the explored pressure range. Within the data accuracy, *B* = 0.088 eV as it changes from 0.0882 to 0.0890 eV in the 0–20 GPa range. We obtain that *C*/*B* = 4.5 in this pressure range.

The present work also provides the dependence of the CF splitting Δ with the crystal volume through *V*(*P*) and *B*(*P*). Figure 7 shows the variation of the CF splitting with *V* and the inset is the variation *B*(*P*) in the studied pressure range. An important conclusion is that, within the accuracy of the data, parameter *B* can be considered as *B* = 0.088 eV in the range 0–20 GPa. In fact, *B* changes only 0.8 meV (from 0.0882 to 0.0890 eV) in this pressure range. In contrast, the variation Δ(*P*) as 8.9 meVGPa^–1^ is plotted as a function of the volume and gives an exponent in the variation as *n* = 3.8(4). This value is smaller than the CF estimates *n* = 5 for an O_h_ symmetry and what has been found for other divalent transition-metal ions in oxides^61^ and even fluorides.^55,62^ The low local symmetry of Ni^2+^ in NiWO_4_ can probably account for the different *n* exponent compared to compounds containing the transition-metal ions in O_h_ sites.

To close this subsection, we would like to comment on finding that a sub-band-gap transition energy (*E*_5_) at the highest pressure of this study becomes the same energy as the fundamental band-gap energy. This fact can be related to the closing of the band gap with pressure in NiWO_4_, which as we discussed before is a typical feature of MWO_4_ orthotungstates when the divalent cation M is a 3*d* element as opposed to the opening of the gap when M is a closed-shell element.^7^

### 3.4 Resistivity and Hall-Effect Experiments

**Figure 8:**
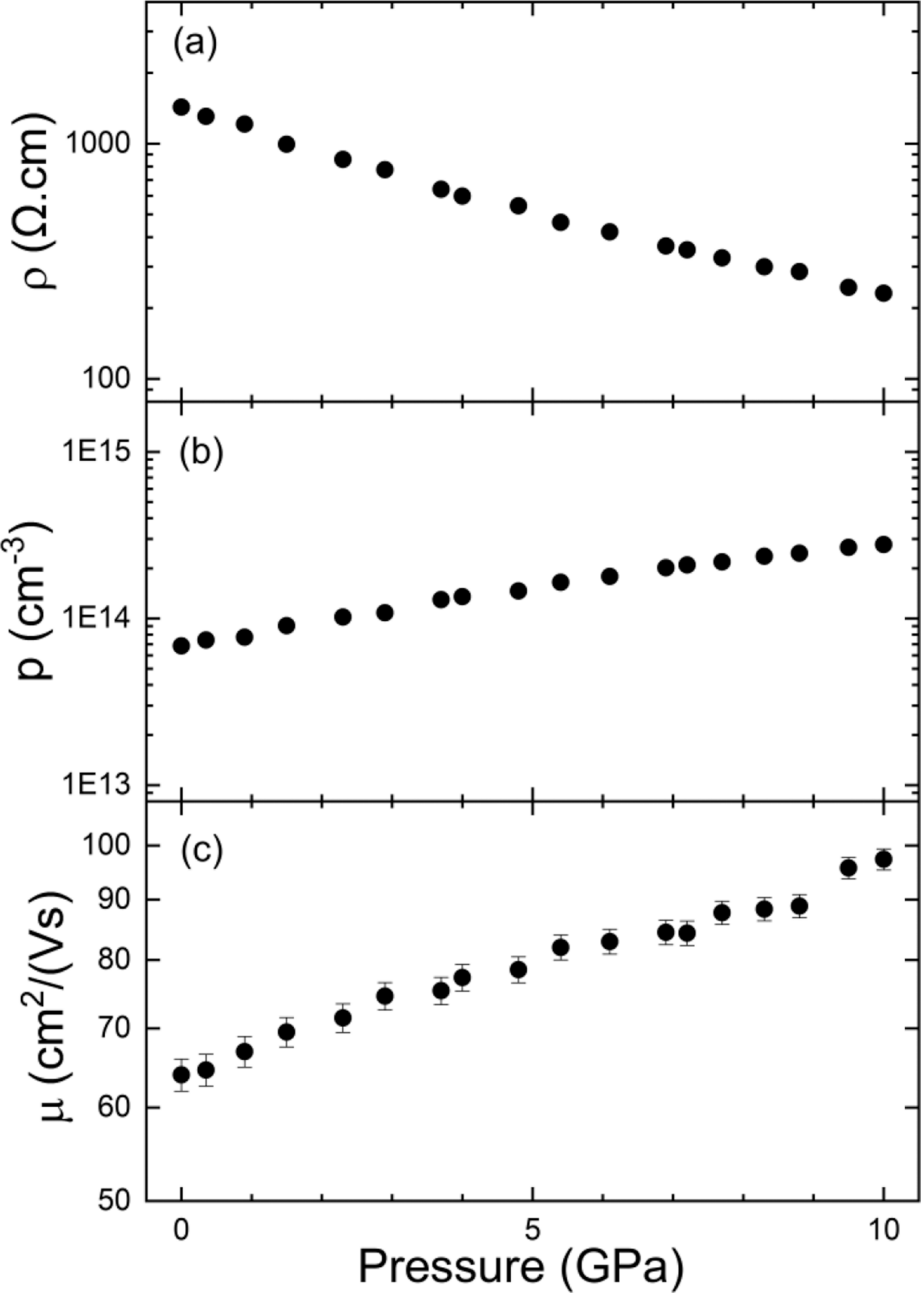
(a) Resistivity, (b) carrier concentration, and (c) carrier mobility of NiWO_4_ versus pressure.

To conclude, we will present results we obtained for electrical transport properties. Our Hall-effect measurements indicate that NiWO_4_ behaves as a *p*-type semiconductor. This could be related with the presence of acceptor levels associated with the presence of Ni vacancies.^63^Figure 8 shows the pressure dependence of the resistivity (ρ), carrier concentration (*p*), and mobility (μ) up to 10 GPa. The resistivity at 0 GPa is comparable with that reported by Bharati et al.^64^ from experiments performed in single crystals. In the past, it was proposed that electrical conductivity can be related to the transfer of *d*-electrons between neighboring metal ions and by exciting electrons from the valence bands to the conduction bands. However, the carrier concentration we measured is more consistent with an extrinsic semiconductor in which related acceptor levels can be caused by a small concentration of Ni vacancies as recently reported in nickel oxide.^63^ Under compression, we found that the resistivity decreases. This is a consequence of both the increase of the carrier concentration and mobility. The increase of the carrier mobility could be related to a decrease of the effective mass of holes, which is consistent with the increase of the convexity of the valence band around the maximum as pressure increases (see Figure 4a,b). On the other hand, assuming the semiconductor is extrinsic, from the pressure dependence of the carrier concentration, we can obtain how the activation energy of donors changes with pressure. From our results, we obtain the activation energy decreases with pressure at a rate of −3.6(3) meV/GPa. Such pressure dependence is comparable to that reported for shallow acceptors in other *p*-type semiconductors.^65,66^ Thus, the presence of acceptors associated with nickel vacancies in NiWO_4_ provides a reasonable hypothesis to explain our transport measurements at ambient and high pressure.

## 4 Conclusions

From powder XRD experiments, we determined the changes induced by pressure in the crystal structure of NiWO_4_. We confirmed that it does not undergo any phase transition up to 20 GPa and determined its bulk modulus, main axes of compressibility, and linear compressibility of these axes. We also determined that NiWO_4_ has a band-gap energy of 3.0 eV which decreases with a pressure coefficient of −13(1) meV/GPa. Both conclusions are supported by DFT calculations which provide a rationale for the changes induced by pressure in the band-gap energy. In particular, calculations show that Ni *3d* electrons play a crucial role in the closing of the band gap. We also demonstrated that the five sub-gap bands of NiWO_4_ correspond to crystal-field transitions within the 3*d*^8^ (*t*_2g_^6^*e*_g_^2^) configuration of Ni^2+^ and clarified their assignment based on their respective energy and pressure shifts. In particular, the different pressure shifts of bands *E*_2_ (0.7 meVGPa^–1^) and *E*_3_ (11.8 meVGPa^–1^) allowed us to unravel their origin as CF transitions to the ^1^*E*_g_(*D*) and ^3^*T*_1g_(*F*) states, respectively, thus resolving a controversial issue in NiWO_4_. Both the energy and pressure dependence were well described on the basis of the semiempirical CF theory using the Tanabe–Sugano method. Finally, resistivity and Hall-effect measurements showed that NiWO_4_ is a *p*-type semiconductor in which the resistivity decreases under compression due to the increase of both the carrier concentration and mobility. This implies that thin films of NiWO_4_ under compressive stress can prove to be better electrode materials for supercapacitors.

## Data Availability

The data that support the findings of this study are available from the corresponding author upon reasonable request.

## Supporting Information Available

Comparisons between measured and calculated energies at ambient pressure and as a function of pressure (PDF)
