# Supplementary material for: Band-Gap Energy and Electronic d–d Transitions of NiWO4 Studied under High-Pressure Conditions
Source: J Phys Chem C Nanomater Interfaces. 2023 Jul 26;127(31):15630–40. doi: 10.1021/acs.jpcc.3c03512 (PMC10426340; doi:10.1021/acs.jpcc.3c03512)
Supplement: Supplementary file 1 — jp3c03512_si_001.pdf [file jp3c03512_si_001.pdf]

Band-gap Energy and Electronic *d-d* Transitions of $NiWO_4$ Studied under High-Pressure Conditions

Daniel Errandonea[1,*], Fernando Rodriguez[2], Rosario Vilaplana[3], David Vie[4], Siddhi Garg[5], Bishnupriya Nayak[5], Nandini Garg[5,6], Jaspreet Singh[7], Venkatakrishnan Kanchana[7], Ganapathy Vaitheeswaran[8]

[1]Departamento de Física Aplicada-ICMUV, MALTA Consolider Team, Universidad de Valencia, Edificio de Investigación, Carrer del Dr. Moliner 50, Burjassot, 46100 Valencia, Spain

[2]DCITIMAC, MALTA Consolider Team, Facultad de Ciencias, Universidad de Cantabria, 39005 Santander, Spain

[3]Centro de Tecnologías Físicas, Universitat Politècnica de València, 46022 Valencia, Spain

[4]Institut de Ciència dels Materials de la Universitat de València, Apartado de Correos 2085, E-46071 València, Spain

[5]High-Pressure and Synchrotron Radiation Physics Division, Bhabha Atomic Research Centre, Trombay, Mumbai 400085, India

[6]Homi Bhabha National Institute, Anushaktinagar, Mumbai 400094, India

[7]Department of Physics, Indian Institute of Technology Hyderabad, Kandi, 502284 Sangareddy, Telangana, India

[8]School of Physics, University of Hyderabad, Prof. C. R. Rao Road, Gachibowli, Hyderabad 500 046, Telangana, India

*Corresponding author: daniel.errandonea@uv.es

Table S1: Comparisons between measured and calculated energies at ambient pressure and as a function of pressure.

P(GPa)	E_i (eV)	E_1	E_2	E_3	E_4	E_5	Bandgap	*B*(eV)	*C*(eV)	Δ(eV)	σ(eV)
Assignment	$^3A_{2g}(F)\rightarrow$	$^3T_{2g}(F)$	$^1E_g(D)$	$^3T_{1g}(F)$	$^1T_{2g}(D)$	$^3T_{1g}(P)$					
0	Exp.	0.95	1.48	1.70	2.40	2.70	3.00				
	Calc.	1.01	1.45	1.64	2.43	2.72		0.088	0.396	1.01	0.04
1.8	Exp.	0.96	1.48	1.72	2.42	2.73	2.96				
	Calc.	1.03	1.45	1.68	2.45	2.74		0.088	0.396	1.03	0.04
3.9	Exp.	0.97	1.48	1.74	2.44	2.76	2.92				
	Calc.	1.04	1.47	1.70	2.48	2.77		0.089	0.400	1.04	0.03
5.6	Exp.	0.99	1.48	1.76	2.45	2.78	2.89				
	Calc.	1.05	1.47	1.70	2.47	2.78		0.089	0.400	1.05	0.04
7.2	Exp.	1.00	1.48	1.77	2.47	2.79	2.86				
	Calc.	1.07	1.44	1.71	2.47	2.78		0.087	0.392	1.07	0.05
8.9	Exp.	1.01	1.48	1.79	2.49	2.81	2.82				
	Calc.	1.08	1.47	1.73	2.50	2.84		0.089	0.400	1.08	0.04
11.7	Exp.	1.03	1.49	1.83	2.50	2.85	2.80				
	Calc.	1.09	1.49	1.75	2.53	2.87		0.090	0.405	1.09	0.05
14.2	Exp.	1.06	1.49	1.87	2.51	2.90	2.72				
	Calc.	1.13	1.47	1.81	2.56	2.91		0.089	0.400	1.13	0.05
16.8	Exp.	1.09	1.49	1.89	2.53	2.93	2.66				
	Calc.	1.15	1.46	1.83	2.57	2.95		0.088	0.396	1.15	0.04
19.6	Exp.	1.13	1.49	1.93	2.55	2.99	2.61				
	Calc.	1.17	1.48	1.85	2.60	2.98		0.089	0.400	1.17	0.04

$C/B = 4.5$; $\sigma = [\Sigma_{i=1-5} [E_i(\text{exp}) - E_i(\text{calc})]^2/5]^{1/2}$
